# Buccal localization of Crohn’s disease with long-term infliximab therapy: a case report

**DOI:** 10.1186/1752-1947-8-397

**Published:** 2014-11-30

**Authors:** Carolina Ciacci, Cristina Bucci, Fabiana Zingone, Paola Iovino, Massimo Amato

**Affiliations:** 6 Gastroenterology Unit, Department of Medicine and Surgery, University of Salerno, Baronissi Campus, via S. Allende, 84081 Baronissi, Salerno Italy; 7 Dentistry Unit, Department of Medicine and Surgery, University of Salerno, Baronissi Campus, via S. Allende, 84081 Baronissi, Salerno Italy

**Keywords:** Cheilitis granulomatosa, Crohn’s disease, Mouth, Infliximab, Treatment

## Abstract

**Introduction:**

Cheilitis granulomatosa causes persistent idiopathic lip swelling and ulceration and it can sometimes be recognized as a unique or early manifestation of Crohn’s disease. Spontaneous remission is rare and with the lack of controlled trials, different therapeutic approaches have been used. Some cases have been treated with an exclusion diet in the attempt to rule out diet allergens, while the most popular treatments include antibiotics such as tetracycline and clofazimine tranilast, benzocaine topical or intralesional steroids, and cheiloplasty, with different outcomes.

**Case presentation:**

We describe the case of a 23-year-old Caucasian man, primarily diagnosed with cheilitis granulomatosa for a severe lower lip swelling, and then with Crohn’s disease of the terminal ileum and anus. Treatment of Crohn’s disease with an anti-tumor necrosis factor alpha agent (infliximab) successfully induced remission of both the gastrointestinal disease and the oral lesion.

**Conclusions:**

Our recommendation is that physicians should be able to recognize cheilitis granulomatosa as a possible marker of a more complex systemic disease and proceed first with an accurate physical examination, and further suggest investigations of the bowel. In cases of Crohn’s disease, a therapy with biological agents can be successful.

## Introduction

Cheilitis granulomatosa (CG) causes persistent idiopathic lip swelling and ulceration and it is included in the orofacial granulomatosis group [1]. The pathogenesis of this disease is still unknown. Spontaneous remission is rare, and with the lack of controlled trials, different therapeutic approaches have been used with regard to the primary etiology of the CG and the personal experience of physicians. CG can occur by itself, can be due to dietary allergens, and also be a feature of Melkersson-Rosenthal syndrome [1]. It can sometimes be recognized as a unique or early manifestation of Crohn’s disease, a disease that may involve the whole gastrointestinal tract, including the mouth and the perianal area [2], although this is considered rare [3]. Here we describe an unusual case of a patient with ileal and perianal Crohn’s disease associated with CG, successfully treated with infliximab.

## Case presentation

**Figure 1:**
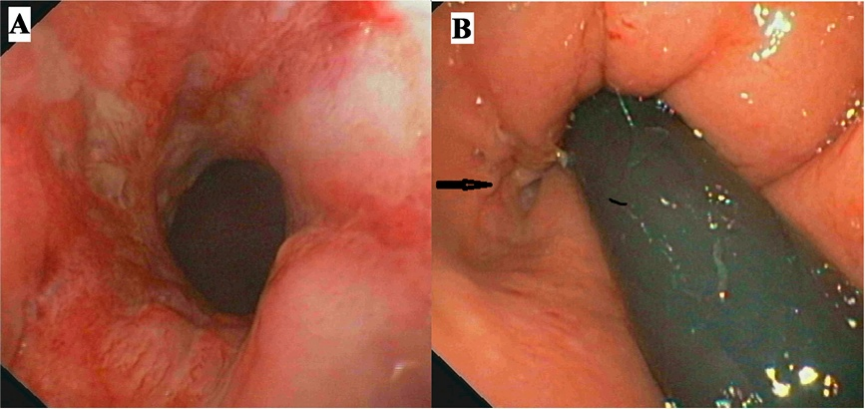
**Colonoscopy. Panel A** shows the rectal inflamed mucosa and **panel B** (arrow) the opening of an anal fistula.

We present the case of a 23-year old-Caucasian man who was initially diagnosed with CG of the lower lip in 2009. He underwent a number of first topic and then systemic treatments with antibiotics and steroids with little or no improvement in the lower lip. In the same period, he underwent psychotherapy for low self-esteem and bad school performance due to his mouth appearance. In January 2011, to confirm the diagnosis of CG, a lower lip biopsy was taken. The pathologist described normal keratinizing squamous epithelium overlying inflammatory tissue with non-caseating granulomatous inflammation in the deeper subcutaneous and parafollicular tissues, consistent with cheilitis granulomatosa. His Ziehl-Neelsen, silver, periodic acid-Schiff, and Warthin–Starry staining results were negative for acid-fast (*Mycobacteria* and *Actinomyces*, specifically), fungal, and spirochetal organisms. In February 2011, he complained of pain during defecation and underwent an evaluation. His rectal examination showed a diffuse, severe, perianal disease characterized by perianal fissures, fistulae, and abscesses. After an in-depth interview, he revealed that in 2009 he had an over-the-counter topic preparation prescribed by his general practitioner (GP) for anal fissuration and had had a moderate discomfort at evacuation since then. The severity of the anal disease and the previous diagnosis of CG alerted us to investigate the possibility of Crohn’s disease by colonoscopy. His endoscopy examination showed a diffuse aphthosis in a very limited region of the rectal ampulla and terminal ileum, and ileal and rectal biopsies were suggestive of a diffuse granulomatous inflammation, compatible with Crohn’s disease (Figure 1). Intestinal ultrasound and magnetic resonance of the intestine confirmed the diagnosis of terminal ileal and perianal Crohn’s disease. After surgical drainage of perianal disease, he was started on infliximab (given as intravenous infusions at dosage of 5mg/kg at 0, 2 and 6 weeks, and at maintenance schedule of 5mg/kg every 8 weeks). He also underwent regular follow-ups that included an endoscopy, histology, and intestinal ultrasound, as per our protocol.

**Figure 2:**
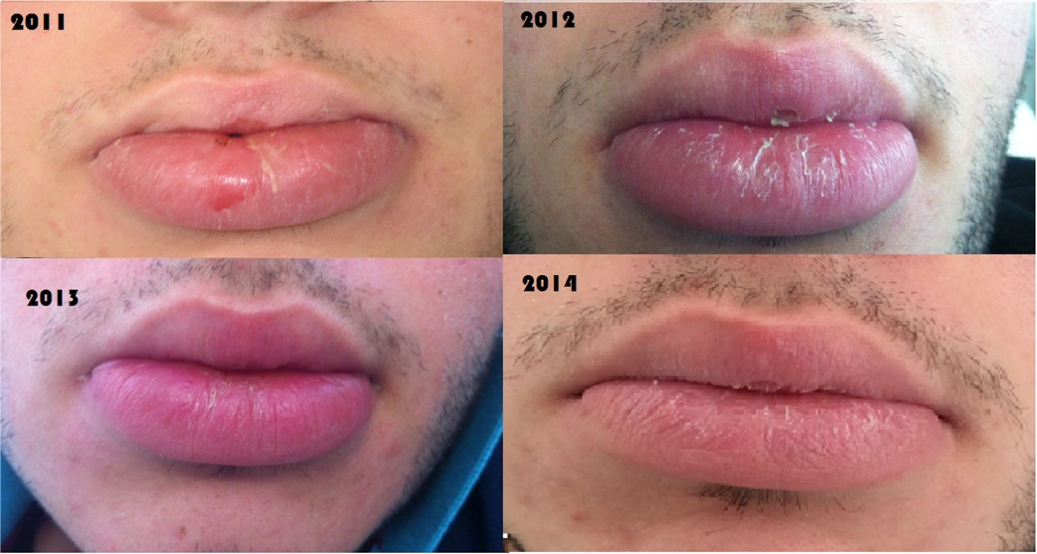
**Modification of lip swelling.** The figure shows gradual changes in lip swelling in our patient since the beginning of infliximab therapy.

Since the beginning of anti-tumor necrosis factor alpha (TNFα) therapy in 2011, we observed a slow decrease of the swelling of his lips as shown in Figure 2, together with a healing of the terminal ileum assessed by endoscopies and magnetic resonance imaging examinations. His perianal lesions disappeared, although a diffuse fibrosis of the anal canal, requiring anal dilations, is still present. No adverse events were noted during the three-year therapy. Notably, the psychological impairment improved and recently he has entered into a nursing school program.

## Discussion

Intestinal Crohn’s disease is accompanied by a number of disease-specific oral lesions, such as swelling of the lips, buccal mucosal swelling or cobble stoning, mucogingivitis, deep linear ulceration, perioral erythema with scaling, recurrent buccal abscesses, and angular cheilitis and mucosal tags [4–6]. These lesions are recurrent and generally improve with immunosuppressant therapy in about 70% of cases [7–10].

It is likely that in our case report Crohn’s disease was already present in 2009 when he was first treated for anal fissure. Concomitantly, the swelling of his lip appeared and diagnosis of CG was made, as described in previous reports [11]. At that time, full attention was paid to the appearance of the mouth and his anal disease was underestimated. Our patient claimed that the CG, more than the anal manifestation, caused the psychological problems and subsequent psychotherapy, as already shown [12]. In our case report, all the Crohn’s disease localizations were successfully treated with infliximab. Also, the psychological impairment was overcome with the healing of his mouth lesion.

Spontaneous remission is rare, and some cases have been treated with an exclusion diet [13]. Table 1 summarizes the most popular treatments of CG including antibiotics such as tetracycline and clofazimine tranilast, benzocaine topical or intralesional steroids, and cheiloplasty, with different outcomes in relation to the follow-up period. Obviously with the lack of controlled trials, different therapeutic approaches have been used with regard to the primary etiology of the CG and the personal experience of physicians.

**Table 1:** **Review of the most relevant literature on treatment of cheilitis granulomatosa**

Author, year of publication	Diagnosis	Number of cases	Therapy	Results
**Martinez Martinez** ***et al*** **. 2012** [14]	CG	6	TCA sulfone, oral steroids, tetracyclines, hydroxychloroquine, amoxicillin-clavulanic acid	Moderate (1 recurrence)
**Ruiz Villaverde and Sanchez, 2012** [15]	CG	1	Adalimumab	Good
**Álvarez-Garrido** ***et al*** **., 2011** [16]	CD + CG	1	Remicade	Good
**Macaigne** ***et al*** **., 2011** [17]	CD + CG	1	Remicade	Good
**Sasaki** ***et al*** **., 2011** [18]	CG	1	Dental treatment	Markedly improved
**Kawakami** ***et al*** **., 2008** [19]	CG	1	Corticosteroid ointment and oral tranilast	None
			Paradentitis treatment	Good
**Mignogna** ***et al*** **., 2008** [9]	CD	1	TCA	Good
**Inui, 2008** [20]	CG	1	Roxithromycin	Good
**Ratzinger** ***et al*** **., 2007** [21]	CG	1	Methylprednisolone, Clofazimine	Moderate
	UG + CD	1	None	None
	MRS	1	Clofazimine	Good
	CG	1	Clofazimine	Good
	CG	1	Clofazimine	None
	CG	1	Clofazimine	Good
	MRS	1	Clofazimine	Good
	BG	1	Clofazimine	Good
	PG	1	Clofazimine	Good
	CG + CD	1	Methylprednisolone, Azathioprine	Good
	MRS	1	Methylprednisolone, Infliximab	Good
	CG	1	None	None
	CG + CD	1	Methylprednisolone, Azathioprine, Infliximab	Good
	PG + CD	1	Clofazimine	Good
**Tonkovic-Capin, 2006** [22]	CD +CG	1	Methotrexate	Good
**Kruse-Losler** ***et al*** **., 2005** [23]	MRS	3	Cheiloplasty	Good
	CG	4		
**Bogenrieder** ***et al*** **., 2003** [24]	CG + CD	1	Mesalazine,	Good
			prednisolone per os	
**Hegarty** ***et al*** **., 2003** [25]	CD + CG	5	Thalidomide	Good
**Sciubba** ***et al*** **.,** **2003** [26]	MRS + oral granulomatosis	7	TCA chlorhexidine,	Moderate
	CD + CG	6	TCA, systemic sulfasalazine/steroids	Moderate
**Kolokotronis** ***et al*** **., 1997** [27]	CG	5	In 3 intralesional corticosteroids	Good
			In 2 oral corticosteroids [2]	
**Ochonisky** ***et al*** **., 1992** [28]	CD	1	Hydroxychloroquine	Good
**Krutchkoff and James, 1978** [29]	CG	1	TCA +cheiloplasty	Good

CG, cheilitis granulomatosa; CD, Crohn’s disease; TCA, triamcinolone acetate intralesional injections; UG, uranitis granulomatosa; PG, pareitis granulomatosa; BG, blepharitis granulomatosa.

Results: none =no remission, moderate =partial remission, good =complete remission. MRS: Melkersson–Rosenthal syndrome.

Orofacial granulomatosis such as CG can be a therapeutic challenge for gastroenterologists and other physicians. When CG is accompanied by a major immune-mediated disease, such as inflammatory bowel disease or arthritis, it is reasonable to consider an anti-TNFα therapy that will control the major disease and improve and/or heal CG. Also, in the case of oral Crohn’s disease, cheiloplasty has not been used so far for fear of surgery complications.

## Conclusions

Orofacial granulomatosis such as CG can be a therapeutic challenge for gastroenterologists and other physicians When CG is accompanied by a major immune-mediated disease, such as inflammatory bowel disease or arthritis, it is reasonable to consider an anti-TNF therapy that will control the major disease and improve and/or heal CG. Also, in the case of oral Crohn’s disease, cheiloplasty has not been used so far for fear of surgery complications.

The patients may indeed psychically suffer from the appearance of their lips, such as in our case report, and request the maximum available therapy. Our experience has demonstrated that infliximab is a useful therapeutic tool for CG associated with Crohn’s disease.

Systematic assessment of the oral cavity will detect the presence of lip swelling, ulcers of the buccal mucosa and sulci, commissures, gingiva, tongue, floor of the mouth, and hard and soft palate and lymph nodes. If oral lesions are present, the next step is to refer the patient to the dentist to photograph, and if needed, take a biopsy of the lesion for follow-up treatment.

Our recommendation is that not only the gastroenterologist but also GPs, dentists and dermatologists, should be able to recognize these lesions as possible markers of a more complex systemic disease and proceed first with accurate physical examination, and further suggest investigation of the bowel.

## Consent

Written informed consent was obtained from the patient for publication of this case report and any accompanying images. A copy of the written consent is available for review by the Editor-in-Chief of this journal.

## Abbreviations

anti-TNFα: Antibodies against tumor necrosis factor alfa
CG: Cheilitis granulomatosa
GP: General practitioner.

## References

[1] Tilakaratne WM, Freysdottir J, Fortune F: **Orofacial granulomatosis: review on aetiology and pathogenesis.** *J Oral Pathol Med* 2008, **37:**191–195. 10.1111/j.1600-0714.2007.00591.x 18321344

[2] Ahmad I, Owens D: **Granulomatous cheilitis and Crohn’s disease.** *Can J Gastroenterol* 2001, **15:**273–275. 10.1155/2001/602183 11331931

[3] Dupuy A, Cosnes J, Revuz J, Delchier JC, Gendre JP, Cosnes A: **Oral Crohn disease: clinical characteristics and long-term follow-up of 9 cases.** *Arch Dermatol* 1999, **135:**439–442. 10.1001/archderm.135.4.439 10206051

[4] Halme L, Meurman JH, Laine P, von Smitten K, Syrjanen S, Lindqvist C, Strand-Pettinen I: **Oral findings in patients with active or inactive Crohn’s disease.** *Oral Surg Oral Med Oral Pathol* 1993, **76:**175–181. 10.1016/0030-4220(93)90200-N 10.1016/0030-4220(93)90200-n 8361727

[5] Issa MA: **Crohn’s disease of the mouth. A case report.** *Br Dent J* 1971, **130:**247–248. 10.1038/sj.bdj.4802654 10.1038/sj.bdj.4802654 5280040

[6] Plauth M, Jenss H, Meyle J: **Oral manifestations of Crohn’s disease. An analysis of 79 cases.** *J Clin Gastroenterol* 1991, **13:**29–37. 10.1097/00004836-199102000-00008 10.1097/00004836-199102000-00008 2007740

[7] Barry O, Barry J, Langan S, Murphy M, Fitzgibbon J, Lyons JF: **Treatment of granulomatous cheilitis with infliximab.** *Arch Dermatol* 2005, **141:**1080–1082. 10.1001/archderm.141.9.1080 10.1001/archderm.141.9.1080 16172303

[8] Gaya DR, Aitken S, Fennell J, Satsangi J, Shand AG: **Anti-TNF-alpha therapy for orofacial granulomatosis: proceed with caution.** *Gut* 2006, **55:**1524–1525. 10.1136/gut.2006.099143 10.1136/gut.2006.099143 PMC1856417 16966709

[9] Mignogna MD, Fortuna G, Leuci S, Amato M: **Oral Crohn’s disease: a favorable clinical response with delayed-release triamcinolone acetonide intralesional injections.** *Am J Gastroenterol* 2008, **103:**2954–2955. 10.1111/j.1572-0241.2008.02094_16.x 19032484

[10] Peitsch WK, Kemmler N, Goerdt S, Goebeler M: **Infliximab: a novel treatment option for refractory orofacial granulomatosis.** *Acta Derm Venereol* 2007, **87:**265–266. 10.2340/00015555-0219 17533496

[11] Ghandour K, Issa M: **Oral Crohn’s disease with late intestinal manifestations.** *Oral Surg Oral Med Oral Pathol* 1991, **72:**565–567. 10.1016/0030-4220(91)90495-X 10.1016/0030-4220(91)90495-x 1745516

[12] Adamo D, Ruoppo E, Leuci S, Aria M, Amato M, Mignogna MD: **Sleep disturbances, anxiety and depression in patients with oral lichen planus: a case-control study.** *J Eur Acad Dermatol Venereol* 2014. doi:10.1111/jdv.12525 10.1111/jdv.12525 24754427

[13] Reed BE, Barrett AP, Katelaris C, Bilous M: **Orofacial sensitivity reactions and the role of dietary components. Case reports.** *Aust Dent J* 1993, **38:**287–291. 10.1111/j.1834-7819.1993.tb05499.x 10.1111/j.1834-7819.1993.tb05499.x 8216037

[14] Martinez Martinez ML, Azana-Defez JM, Perez-Garcia LJ, Lopez-Villaescusa MT, Rodriguez Vazquez M, Faura BC: **Granulomatous cheilitis: a report of 6 cases and a review of the literature.** *Actas Dermosifiliogr* 2012, **103:**718–724. 10.1016/j.ad.2012.02.005 10.1016/j.ad.2012.02.005 22657098

[15] Ruiz Villaverde R, Sanchez CD: **Successful treatment of granulomatous cheilitis with adalimumab.** *Int J Dermatol* 2012, **51:**118–120. 10.1111/j.1365-4632.2010.04507.x 10.1111/j.1365-4632.2010.04507.x 22182390

[16] Alvarez-Garrido H, Pericet-Fernandez L, Martinez-Garcia G, Tejerina-Garcia JA, Peral-Martinez I, Miranda-Romero A: **Crohn’s disease and cheilitis granulomatosa: role of silicone fillers.** *J Am Acad Dermatol* 2011, **65:**239–241. 10.1016/j.jaad.2009.12.055 10.1016/j.jaad.2009.12.055 21679838

[17] Macaigne G, Harnois F, Boivin JF, Dikov D, Ridoux G, Cheaib S, Chayette C: **Crohn’s disease revealed by a cheilitis granulomatosa with favorable evolution by perfusions of infliximab: report of a case and review of the literature.** *Clin Res Hepatol Gastroenterol* 2011, **35:**147–149. 10.1016/j.clinre.2010.10.001 10.1016/j.clinre.2010.10.001 21809492

[18] Sasaki R, Suzuki K, Hayashi T, Inasaka H, Matsunaga K: **Improvement of cheilitis granulomatosa after dental treatment.** *Case Rep Dermatol* 2011, **3:**151–154. 10.1159/000330731 10.1159/000330731 PMC3177833 21941479

[19] Kawakami T, Fukai K, Sowa J, Ishii M, Teramae H, Kanazawa K: **Case of cheilitis granulomatosa associated with apical periodontitis.** *J Dermatol* 2008, **35:**115–119. 10.1111/j.1346-8138.2008.00426.x 10.1111/j.1346-8138.2008.00426.x 18271809

[20] Inui S, Itami S, Katayama I: **Granulomatous cheilitis successfully treated with roxithromycin.** *J Dermatol* 2008, **35:**244–245. 10.1111/j.1346-8138.2008.00455.x 10.1111/j.1346-8138.2008.00455.x 18419685

[21] Ratzinger G, Sepp N, Vogetseder W, Tilg H: **Cheilitis granulomatosa and Melkersson-Rosenthal syndrome: evaluation of gastrointestinal involvement and therapeutic regimens in a series of 14 patients.** *J Eur Acad Dermatol Venereol* 2007, **21:**1065–1070. 10.1111/j.1468-3083.2007.02194.x 10.1111/j.1468-3083.2007.02194.x 17714126

[22] Tonkovic-Capin V, Galbraith SS, Rogers RS III, Binion DG, Yancey K: **Cutaneous Crohn’s disease mimicking Melkersson-Rosenthal syndrome: treatment with methotrexate.** *J Eur Acad Dermatol Venereol* 2006,**20**(4)**:**449–452. 10.1111/j.1468-3083.2006.01458.x 10.1111/j.1468-3083.2006.01458.x 16643147

[23] Kruse-Losler B, Presser D, Metze D, Joos U: **Surgical treatment of persistent macrocheilia in patients with Melkersson-Rosenthal syndrome and cheilitis granulomatosa.** *Arch Dermatol* 2005, **141:**1085–1091. 10.1001/archderm.141.9.1085 16172304

[24] Bogenrieder T, Rogler G, Vogt T, Landthaler M, Stolz W: **Orofacial granulomatosis as the initial presentation of Crohn’s disease in an adolescent.** *Dermatology* 2003, **206:**273–278. 10.1159/000068900 10.1159/000068900 12673090

[25] Hegarty A, Hodgson T, Porter S: **Thalidomide for the treatment of recalcitrant oral Crohn’s disease and orofacial granulomatosis.** *Oral Surg Oral Med Oral Pathol Oral Radiol Endod* 2003, **95:**576–585. 10.1067/moe.2002.113 10.1067/moe.2002.113 12738949

[26] Sciubba JJ, Said-Al-Naief N: **Orofacial granulomatosis: presentation, pathology and management of 13 cases.** *J Oral Pathol Med* 2003, **32:**576–585. 10.1034/j.1600-0714.2003.t01-1-00056.x 10.1034/j.1600-0714.2003.t01-1-00056.x 14632932

[27] Kolokotronis A, Antoniades D, Trigonidis G, Papanagiotou P: **Granulomatous cheilitis: a study of six cases.** *Oral Dis* 1997, **3:**188–192. 10.1111/j.1601-0825.1997.tb00034.x 9467364

[28] Ochonisky S, Bonvalet D, Caron C, Kornhauser R, Vignon-Pennamen MD, Dubertret L: **Granulomatous cheilitis with cutaneous extension in Crohn disease. Regression with hydroxychloroquine.** *Ann Dermatol Venereol* 1992, **119:**844–846. 1301697

[29] Krutchkoff D, James R: **Cheilitis granulomatosa. Successful treatment with combined local triamcinolone injections and surgery.** *Arch Dermatol* 1978, **114:**1203–1206. 10.1001/archderm.1978.01640200057015 10.1001/archderm.114.8.1203 677920

